# A Lycopene ε-Cyclase TILLING Allele Enhances Lycopene and Carotenoid Content in Fruit and Improves Drought Stress Tolerance in Tomato Plants

**DOI:** 10.3390/genes14061284

**Published:** 2023-06-17

**Authors:** Angelo Petrozza, Stephan Summerer, Donato Melfi, Teresa Mango, Filippo Vurro, Manuele Bettelli, Michela Janni, Francesco Cellini, Filomena Carriero

**Affiliations:** 1 ALSIA Centro Ricerche Metapontum Agrobios, s.s. Jonica 106, km 448.2, 75010 Metaponto, MT, Italy; angelo.petrozza@alsia.it (A.P.); stephan.summerer@alsia.it (S.S.); donato.melfi@alsia.it (D.M.); teresa.mango@alsia.it (T.M.); francesco.cellini@alsia.it (F.C.); 2 Istituto dei Materiali per l’Elettronica e il Magnetismo (IMEM-CNR), Parco Area delle Scienze 37/A, 43121 Parma, Italy; filippo.vurro@imem.cnr.it (F.V.); manuele.bettelli@imem.cnr.it (M.B.); michela.janni@imem.cnr.it (M.J.)

**Keywords:** *Solanum lycopersicum*, induced mutations, carotenoid pathway, abiotic stress, digital image-based phenotyping, bioristor

## Abstract

In the scenario of climate change, the availability of genetic resources for tomato cultivation that combine improved nutritional properties and more tolerance to water deficiency is highly desirable. Within this context, the molecular screenings of the Red Setter cultivar-based TILLING platform led to the isolation of a novel lycopene ε-cyclase gene (SlLCY-E) variant (G/3378/T) that produces modifications in the carotenoid content of tomato leaves and fruits. In leaf tissue, the novel G/3378/T SlLCY-E allele enhances β,β-xanthophyll content at the expense of lutein, which decreases, while in ripe tomato fruit the TILLING mutation induces a significant increase in lycopene and total carotenoid content. Under drought stress conditions, the G/3378/T SlLCY-E plants produce more abscisic acid (ABA) and still conserve their leaf carotenoid profile (reduction of lutein and increase in β,β-xanthophyll content). Furthermore, under said conditions, the mutant plants grow much better and are more tolerant to drought stress, as revealed by digital-based image analysis and in vivo monitoring of the OECT (Organic Electrochemical Transistor) sensor. Altogether, our data indicate that the novel TILLING SlLCY-E allelic variant is a valuable genetic resource that can be used for developing new tomato varieties, improved in drought stress tolerance and enriched in fruit lycopene and carotenoid content.

## 1. Introduction

Tomato is one of the most widely consumed vegetables in the world. Tomato plants are sensitive to lack of water during reproductive development, especially during flowering and fruit growth [1]. Under drought stress conditions, tomato plants exhibit reduced leaf area, reduced growth, flower drop, mineral deficiency, reduced fruit size, fruit breakage, and calcium deficiency-related physiological disorders, such as flower rot and poor seed viability [2]. Therefore, improvement in the drought resistance of tomato varieties is a focus for researchers and breeders [3].

Under water-deficit conditions, plant cells perceive stress through cell membrane receptor/sensors’ activity [3]. Following this, a range of second messengers, such as abscisic acid (ABA), inositol phosphate, calcium ions, reactive oxygen species (ROS), transcriptional regulators, and phosphoinositides, are activated [4]. Subsequently, plants trigger a series of physiological defence responses in order to reduce water loss by partial stomatal closure, controlled water transpiration, and modulation of the expression level of several drought-perceptive genes [5].

Tomatoes are also one of the principal dietary sources of antioxidants, such as carotenoids, tocopherols and flavonoids. Among carotenoids, which are the most abundant antioxidants, ripe tomato fruits mainly accumulate lycopene. Lycopene is a C40 compound that is synthesized via carotenoid metabolism during fruit ripening [6]. The cyclization of lycopene is an important branching point in the carotenoid biosynthesis pathway (Figure S1). In one route, lycopene ε-cyclase, together with lycopene β-cyclase, makes α-carotene and, consequently, lutein. In the alternative route, lycopene β-cyclase alone leads to the formation of β-carotene and its derivative xanthophylls [6]. The β,β xanthophyll, 9-cis-violaxanthin and 9-cis-neoxanthin are the precursors for the synthesis of abscisic acid (ABA), the plant hormone involved in the response to abiotic stress [7,8].

Lycopene is a strong antioxidant and has been shown to be able to reduce the risk of prostate cancer [9] and cardiovascular diseases [10]. The important health benefits of lycopene have prompted studies aimed at enhancing lycopene accumulation to improve the nutraceutical properties of tomato fruit.

Over-expression or down-regulation of genes encoding key enzymes of the lycopene biosynthesis pathways are the most effective approaches to increasing lycopene content [11,12,13]. Modulation of the chromoplast-specific lycopene β-cyclase (Cyc-B) enzyme by means of a TILLING missense point mutation was also successful in incrementing the lycopene content in tomato fruit [14]. Recently, Xindi Li et al. [15] used the CRISPR/Cas9 system to increase lycopene level in tomato fruit via the concurrent inhibition of multiple genes of the carotenoid pathway.

In diverse plant species, genes/enzymes involved in the conversion of lycopene to β- and α-carotene are often the main targets in incrementing total carotenoids. In *S. lycopersicum*, the overexpression of tomato lycopene β-cyclase cDNA increased the total carotenoid content in fruits, in addition to the almost complete conversion of lycopene into β-carotene [16]. The down-regulation of the lycopene ε-cyclase gene using RNAi increased the total carotenoid content of *Brassica napus* seeds [17]. In sweet potato, *Ipomoea batatas*, the increment in carotenoid level in storage roots and leaves was obtained both by reducing the expression of lycopene ε-cyclase gene [18] and by overexpression of a new allele of the lycopene β-cyclase (*IbCYB2*) gene [19]. With the modulation of lycopene ε-cyclase and lycopene β-cyclase genes, along with carotenoid enrichment, an increase in abiotic stress tolerance was also observed in sweet potato.

Nowadays, in the scenario of climate changes and water scarcity, tomato germplasm that combines improved nutritional properties and increased capability to face adverse conditions is highly demanded by breeders.

With the aim of increasing the availability of tomato genetic resources improved in fruit carotenoid content and abiotic stress tolerance, we exploited TILLING (Targeting Induced Local Lesions IN Genomes) [20] technology to select new alleles in the tomato Red Setter mutant population [21].

We searched for new allelic variants of the lycopene ε-cyclase gene which, in sweet potato plants down-regulated via RNAi, resulted in increased total carotenoid content and abiotic stress tolerance [18]. The search for new SlLCY-E alleles by TILLING molecular screenings led to the isolation of a novel lycopene ε-cyclase (SlLCY-E) variant containing a point mutation (G/3378/T) with potential enhancement effects on lycopene and total carotenoid biosynthesis in ripe tomato fruit and on β,β-xanthophyll increase in leaf tissue.

The evaluation of underwater deficiency of the G/3378/T LCY-E allele via in vivo monitoring of the OECT sensor [22] and digital-based images showed that the G/3378/T LCY-E plants are more tolerant to drought stress. Altogether, our data demonstrate that the G/3378/T LCY-E allele is a valuable and promising genetic resource, useful for developing new tomato varieties with improved drought stress tolerance features, and enriched in fruit lycopene and carotenoid content.

## 2. Materials and Methods

### 2.1. Plant Material

In this work the following tomato (*S. lycopersicum*) lines were used: Red Setter, referred to as WT (wild type), which is an old processing tomato cultivar, and the EMS-generated mutant lyc-e (referred to as M, mutant), selected through the TILLING approach, from the Red Setter mutant collection [21]. Plant material was grown in a greenhouse under environmentally controlled conditions at a temperature of 22 °C with a photoperiod of 16 h light/8 h dark.

### 2.2. Mutant Allele Identification

The search for induced point mutations in the *SlLCY-E* gene (Solyc12g008980.1) was done in the Red Setter TILLING platform [21] according to the experimental conditions described in Dalmais et al. [23]. The Red Setter *SlLCY-E* mRNA sequence, deposited in Genbank database (https://www.ncbi.nlm.nih.gov/, accessed on 27 April 2020) under the accession number EU533951.1, was used to retrieve the *SlLCY-E* gene structure information in the SGN website (https://solgenomics.net/, accessed on 27 April 2020) and the genomic sequence then used for primer design. Two couples of *SlLCY-E* gene-specific primers were selected and employed in the molecular screening based on nested-PCR (Table 1).

The first PCR was conducted with the external primers (Fw-ext/Rev-ext) and the second PCR with the internal forward (Fw-int) and reverse (Rev-int) primers 5′-end labelled with IRDye 700 and IRDye 800 dye (LI-COR^®^, Lincoln, NE, USA), respectively.

The detection of mutations was performed with the mismatch specific endonuclease ENDO I [24] and the LI-COR 4300 DNA analyzer (LI-COR^®^, Lincoln, NE, USA). The information on the identity and nucleotide change position in the screened region was obtained via Sanger sequence analysis.

### 2.3. Drought Stress Experiments

Two drought stress experiments were performed with the lcy-e mutant line and the Red Setter control genotype: the first one in summer and the second one in autumn season. The drought stress effects on tomato plants were studied with different approaches in the two seasons: in summer, the plants were subjected to biochemical analysis, and in autumn they were pheno-typed via digital images and in vivo monitoring of the bioristor. The summer experiment took place during the last two weeks of July under unregulated conditions. In a greenhouse with an average daily temperature of 30 °C, a relative humidity of 45% and solar radiation of 167 Par., 24 plants of Red Setter control genotype and 24 plants of lcy-e were grown in 16 cm diameter soil-filled pots and fully irrigated until the 5th–6th true leaf stage.

At this development stage, for each genotype, 12 plants were irrigated with 250 mL of water twice daily, early in the morning and in the evening, while 12 plants were exposed to drought stress by withholding watering for 15 days.

Before the drought stress imposition, leaf material was collected from mutant and control tomato genotypes (day 0) and from drought stressed (DS) and well-watered (WW) plants at 3, 6 and 10 days after stress application. On the 15th day of the stress imposition, water was restored and two hours after the irrigation leaf material was collected.

For each sampling day, leaf material was harvested at the same time (9.00 a.m.) and for each genotype and for each experimental condition (stress and no stress application) leaf tissue was harvested from 3 independent plants (3 biological replicates). The sampled green material was immediately frozen and stored at −80 °C then lyophilized and used in the carotenoid and ABA measurement analysis.

In autumn, the drought stress experiment was conducted in the greenhouse under controlled conditions. The experiment involved the use of eight replicas per genotype and per treatment. The plants were grown in 16 cm diameter soil-filled pots, exposed to a photoperiod of 12 h (light intensity 180 Par) with a daytime temperature of 22 °C and a night-time temperature of 16 °C; the relative humidity ranged from 50 to 60%.

**Figure 1:**
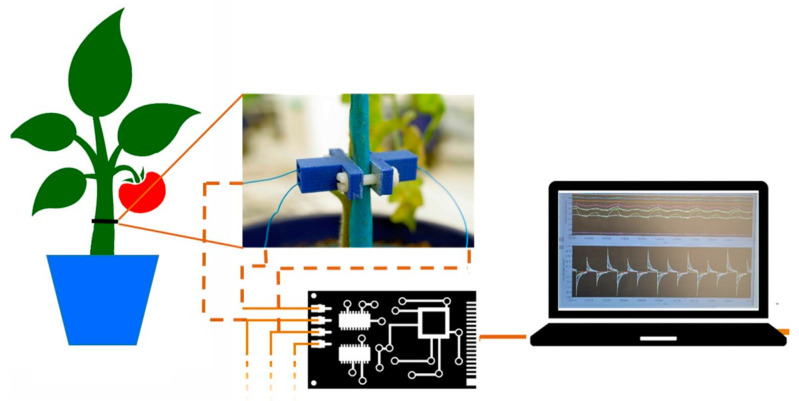
Scheme of tomato monitoring through bioristor: an in vivo phenotyping approach.

When the plants reached the 5th leaf expansion phase, one bioristor sensor was inserted in each plant (Figure 1). Plants were kept fully irrigated for 1 day post insertion, and drought stress was applied for 13 days. Six plants of each genotype were subjected to the suspension of irrigation while six plants were kept normally irrigated as control.

### 2.4. Carotenoid Analysis

The carotenoid analyses were conducted as previously described in D’Ambrosio et al. [25], with the Agilent 1200 Chemstation HPLC system (Agilent Technologies, Milano, Italy) and a C-30 4,6x250 mm reversed phase column (YMC Europe GmbH, Dinslaken, Germany). The pigments were extracted from leaves and ripe fruits and the assays were carried out with at least three biological replicates.

Carotenoids were identified and quantified using calibration curves of the standard compounds of violaxanthin, neoxanthin, supplied by Carotenature GmbH (Carotenature GmbH, Bern, Switzerland), lutein, zeaxanthin, b-carotene, and lycopene e 8′-apo-β-carotenal (internal standard) from Sigma Aldrich (Merk KGaA, Darmstadt, Germany).

### 2.5. ABA Measurement

The leaf ABA content was determined by LC-MS analysis according to the protocol reported in Rong Zhou et al. [26]. The analyses were performed on an Agilent 1290 infinity series (Agilent Technologies, Milano, Italy) equipped with a Model G4220A binary pump, G6410B mass detector, G4226A autosampler and a G1316C column compartment (Agilent Technologies, Milano, Italy). All mass spectra were obtained by Mass Hunter workstation data acquisition software (v B.08.02, Agilent Technologies, Milano, Italy) and were analyzed by Mass Hunter workstation software (v B.08.02, Agilent Technologies, Milano, Italy) for qualitative and quantitative analysis.

### 2.6. Leaf Stomatal Conductance

Leaf stomatal conductance was performed on two fully expanded leaves from each plant. Measurements were taken on a central portion of leaves (fourth and fifth leaf from the ground) using a steady-state Leaf Porometer (SC-1 Decagon Devices, Pullman, WA, USA).

### 2.7. Digital Imaging Phenotyping: Data Acquisition and Processing

RGB images were captured using a Scanalyzer 3D platform (LemnaTec GmbH, Aachen, Germany), described in Marko et al. [27]. These result in three mutually orthogonal views which were used to assess plant morphometric parameters, such as height and biomass (biovolume). The digital biovolume (projected shoot area, PSA) was calculated as the sum of the projected plant area from the three orthogonal images of the same plant. This digital trait is used as a digital proxy for fresh weight. Solidity, which indicates how much of the hull area is covered by leaves, is calculated as the projected shoot area/convex hull area from plant images. A measure of the plant color was determined by calculating the weighted mean value from the histogram of the hue channel in the HSV color space resulting in a value from 0–360°, where yellow and green are respectively at 60° and 120°, yellow indicating chlorotic tissue and green healthy tissue.

### 2.8. Bioristor Measurements

Bioristor is an Organic Electrochemical Transistor (OECT) based sensor composed of a channel and gate electrodes, both constituted by a textile fiber functionalized with a conductive polymer [28,29] and directly integrated into the plant stem (Figure 1). For the functionalization, two textile fibers were soaked for 5 min in aqueous poly(3,4-ethylenedioxythiophene) doped with polystyrene sulphonate (CleviosPH500, Starck GmbH, Munich, Germany), after which ethylene glycol (10% *v*/*v*) and dodecyl benzene sulphonic acid (2% *v*/*v*) were added. Fibers were baked at 130 °C for 90 min. Before functionalization, each thread was cleaned with a plasma oxygen cleaner treatment (Femto, Diener electronic, Ebhausen, Germany) to increase its wettability and facilitate the adhesion of the aqueous conductive polymer solution. Twice treated fiber was completely inserted into the stem of each tomato plant, as channel and gate of the OECT.

The fiber was connected at each end to a metal wire with silver paste to stabilize the connections, forming the “source” and “drain” electrodes. The transistor device was completed by introducing a second functionalized textile thread as the gate electrode [22].

Bioristors are connected to a digital converter board NI USB-6343 multifunction I/O device (National Instruments, Austin, TX, USA). Drain and gate currents play a major role in determining the sensor response. The p-type doped PEDOT (oxidized from the electrochemistry point of view) leads to mobile holes generating a hole current (I_ds0_) which flows in the channel when a drain voltage (V_ds_ = −0.05 V) is applied. These holes are balanced by the negative charge of the PSS sulphonate group [30] until the application of a positive gate bias (V_g_ = 0.6 V), which leads to the injection of cations (M^+^) from the electrolyte (xylem sap in this case) into the PEDOT:PSS channel, causing its de-doping, according to equation [31].

(1) $${PEDOT}^{+}:{PSS}^{-}+M^{+}+e^{-}\to {PEDOT}^{0}+M^{+}:{PSS}^{-}$$

The “de-doping process” [32], according to the reduction of the oxidized PEDOT^+^ to PEDOT^0^ and the decrease of the number of holes in the channel, leads to a drop in the drain current (I_ds_). The whole process is reversible: when gate-source voltage is switched off (V_g_ = 0 V), cations diffuse from the channel to the electrolyte, increasing the number of conducting holes and, consequently, reduced PEDOT^0^ returns to the oxidized state and drain-source current to the initial value (I_ds0_).

The sensor response (R) can be expressed as

(2) $$R=\frac{\left|I_{ds}-I_{ds0}\right|}{I_{ds0}}$$

and is related to the cation concentration in the electrolyte solution [29], thus allowing the monitoring of the temporal variation in the plant sap’s cationic content. Here, R measured in stressed plants (R_stress_) and in control plants (R_control_) is reported.

When monitoring the plant sap concentration over several days, it proved useful to smooth out the day/night signal oscillations due to plant circadian rhythms that characterize bioristor response [28]. The ratio between the signal recorded from sensors installed in water-stressed plants and control plants was expressed as Normalized Response [22]:

(3) $$NR=\frac{R_{stress}}{R_{control}}$$

### 2.9. Statistical Analysis

For carotenoid content and phenomics work, statistical analysis was performed using the base functions of the R software (4.2 version). One-way ANOVA was used to examine differences in the means and standard error of carotenoid content, ABA content and plant phenomics data. From this ANOVA analysis, significant differences were identified with the TukeyHSD post-hoc test. The bioristor R index data were subjected to analysis using MATLAB (MathWorks, Natick, MA, USA) and Microsoft Excel 2016 to address variations related to the circadian cycle [22]. Specifically, a rolling mean calculation was employed to smoothen the data, thereby reducing background noise and enhancing the visibility of variations linked to drought occurrences. For statistical analysis of the R data, Analysis of Variance (ANOVA) was conducted using MATLAB (MathWorks, Natick, MA, USA), with the resulting *p*-value being computed. Additionally, Principal Component Analysis (PCA) was performed using the R statistical analysis software function “prcomp,” and the findings were visualized through a biplot generated with the “factoextra” package. The biplot effectively summarized the first two principal components, PC1 and PC2, along with their respective component loading vectors. Furthermore, the component scores were depicted as colored dots, providing classification information based on the thesis classification scheme [33].

## 3. Results

### 3.1. Discovery and Phenotyping of a Novel SlLCY-E Allele

**Figure 2:**
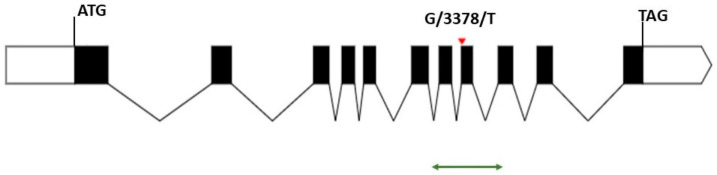
Scheme of SlLCY-E genomic sequence (Solyc12g008980.1). The lcy-e missense mutation is highlighted by a red triangle and its position, G/3378/T, is indicated relative to the first nucleotide of the start codon of the SlLCY-E genomic sequence (4956 bp long from ATG to TAG). The dark boxes represent exons codifying for the 527 aa of the LCY-E protein while the thin lines represent introns. The green arrow indicates the 684 bp region screened by TILLING. The SlLCY-E gene structure was determined using Exon-Intron Graphic Maker (http://wormweb.org/exonintron, accessed on 9 May 2022).

The search for induced point mutations in lycopene ε-cyclase gene (SlLCY-E, Solyc12g008980.1) was carried out on the tomato Red Setter cultivar-based TILLING platform [21]. The molecular screening of a 684 bp region, encompassing exon 7, exon 8 and 51 bp of exon 9, of SlLCY-E gene (Figure 2), allowed the identification of a missense mutation (G/3378/T), causing the amino acid change of Tryptophan with Leucin (W/356/L) at position 356 of the lycopene ε-cyclase protein.

Since lycopene ε-cyclase is a key enzyme in the carotenoid biosynthesis pathway, we first analyzed the leaf and fruit carotenoid content of M4 lcy-e plants (M, mutant), homozygous for the discovered point mutation, to verify whether the amino acid change W/356/L could affect the plant phenotype.

In the phenotypic characterization, the non-mutagenized plants of Red Setter (WT, wild type) were used as control.

The results of leaf and ripe fruit pigment analysis showed significant differences in lcy-e mutant with respect to the Red Setter control line (Table 2). In the leaf tissue of lcy-e plants the violaxanthin content was double that of Red Setter (289 vs. 144 µg/g of dry weight, DW), while for its precursor, zeaxanthin, an increment of 30% with respect to the wild type line was measured. A significant decrease in lutein amount was instead observed in leaves (397 vs. 615 µg/g DW of the WT) and ripe fruits of the mutant line (7.19 vs. 20.60 µg/g DW of the WT). In addition to the lutein content reduction, the HPLC determinations of carotenoids in ripe fruit showed an increase of lycopene (23%) and total carotenoid (19%). The total carotenoid content, as well as the lycopene content of lcy-e fruit, were significantly different from the Red Setter control fruit.

Altogether, the leaf and fruit carotenoid results demonstrated that the G/3378/T missense mutation, present in the lcy-e line, is able to produce metabolic phenotypic effects.

### 3.2. First Drought Stress Experiment

#### 3.2.1. Analysis of Leaf Carotenoid Content

Both 9-cis-violaxanthin and 9-cis-neoxanthin have been proposed to be the precursors for abscisic acid biosynthesis, the key hormone involved in the plant response to abiotic stress and, in particular, to drought stress. Since the HPLC determinations of leaf carotenoids revealed a higher content of violaxanthin in lcy-e mutant plants with respect to the control line Red Setter, we tested the G/3378/T LCY-E allele in a suspended irrigation regime with the aim of evaluating its response towards adverse conditions, such as that produced by the drought stress.

**Figure 3:**
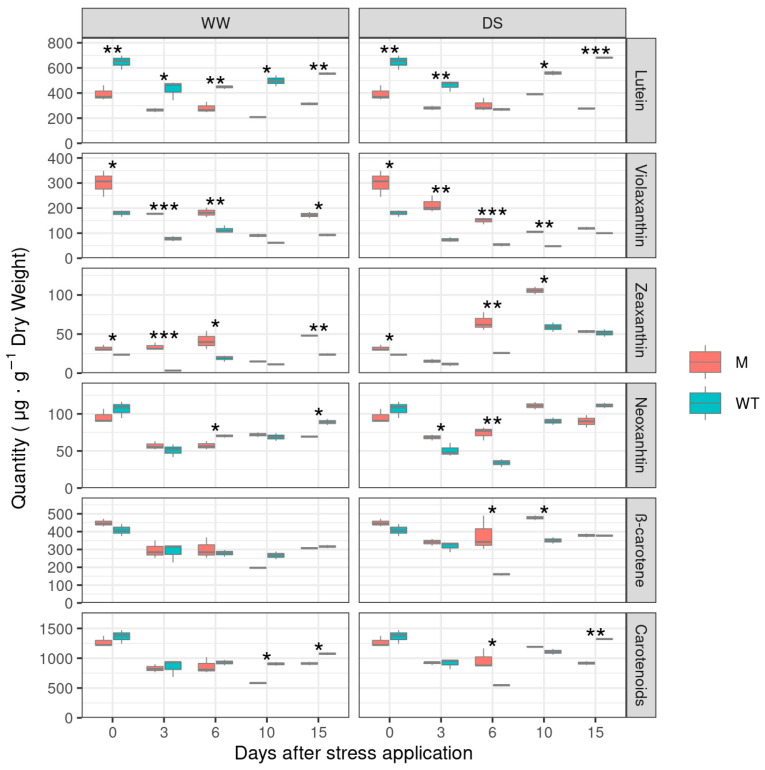
Leaf carotenoid content of WW and DS lcy-e (M) and Red Setter plants (WT). The HPLC determinations of carotenoids were carried out before the drought stress imposition (day 0), at 3, 6, 10 days after the stress application and two hours later than the restored water (day 15). Values are the mean of three biological replicates ±SD. A one-way ANOVA was used to examine the differences between genotypes at each timing point; the differences among means were identified by Tukey Post-Hoc Test; asterisks indicate significance (* *p* < 0.05, ** *p* < 0.01 *** *p* < 0.001).

The first drought stress experiment was conducted with the lcy-e mutant line and the wild type Red Setter during the summer, as reported in Materials and Methods. To determine whether the plant response to the stress imposition was correlated with alterations in the carotenoid biosynthesis, well-watered (WW) and drought stressed (DS) Red Setter and lcy-e plants were analyzed for their leaf carotenoid content at 0, 3, 6, 10 and 15 days (Figure 3).

Concerning lutein content, it was significantly higher in the Red Setter control line than the mutant genotype, irrespective of watering conditions at all the timing points, except for the 6 day point, where similar concentrations were measured in DS plants of M and WT. Lutein content was roughly doubled in Red Setter compared to lcy-e plants. On the contrary, violaxanthin content was, on average, double that in the mutant than in wild type, both in WW and DS plants, at 0, 3, 6, 10 and 15 days.

A significant difference in zeaxanthin content was also registered between the mutant line and the control genotype, both in WW and DS plants. The violaxanthin precursor was higher in lcy-e plants at all analyzed time points, except for the lcy-e WW plants at 10 days and the lcy-e DS plants at 3 and 15 days, where the recorded amounts were similar in both genotypes.

The neoxanthin content was similar in the two genotypes before the drought stress imposition (day 0), it decreased at 3 and 6 days in WW and DS plants of both genotypes, while at 10 and 15 days it started to increase, reaching values similar to those registered at the beginning of the experiment (day 0). A significantly higher content of neoxanthin was observed in Red Setter WW plants at 6 days, while under stress conditions, at the same time point, the neoxanthin amount was higher in DS lcy-e plants than DS Red Setter plants. A significantly higher neoxanthin content was also measured in lcy-e DS plants at 3 days after stress imposition.

Regarding β-carotene, no significant differences were registered between the two WW genotypes at all the analyzed time points. On the contrary, a higher content of β-carotene was observed in DS lcy-e plants at 6 and 10 days.

The total carotenoid content was, on average, similar in WW and DS plants of Red Setter and lcy-e lines except at 6 days, when in the lcy-e DS plants it was double that measured in Red Setter DS plants, while an opposite trend was observed at 10 and 15 days for the wild type control line.

Overall, the leaf HPLC determinations of WW and DS plants confirmed the lcy-e carotenoid profile previously observed, i.e., reduction of lutein and increase of violaxanthin and zeaxanthin content with respect to the control line Red Setter.

#### 3.2.2. ABA Measurements

**Figure 4:**
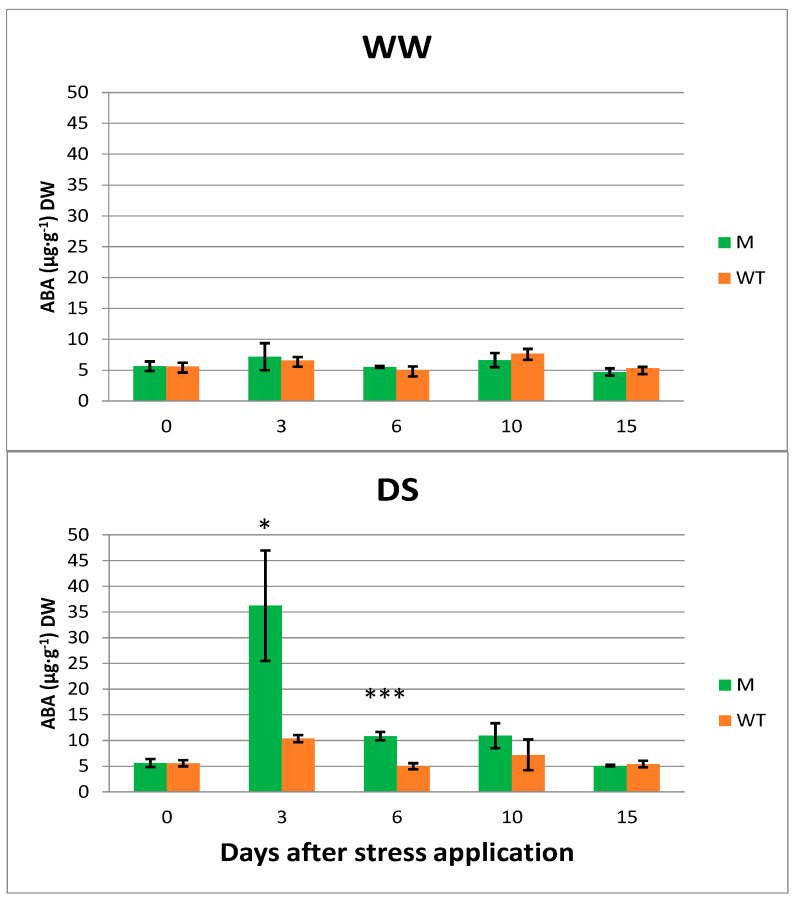
Quantitative analysis of ABA content in leaves of WW and DS lcy-e (M) and Red Setter plants (WT). The ABA levels were taken before the drought stress imposition (day 0), at 3, 6 10 days after stress application and two hours later than the restored water (day 15). Values are the average of three biological replicates ±SE. A one-way ANOVA was used to examine the differences between genotypes at each timing point; the differences among means were identified by Tukey Post-Hoc Test; asterisks indicate significance (* *p* < 0.05, *** *p* < 0.001).

Well-watered and drought stressed plants of lcy-e mutant and Red Setter control genotypes were also analyzed for their ABA content by LC-MS (Figure 4).

No significant differences in ABA content were observed between WW lcy-e and Red Setter plants at all the analyzed time points. Conversely differences in abscisic acid amounts were detected between DS lcy-e and Red Setter plants. Before the start of stress imposition (day 0), the ABA content was similar in both genotypes. After three days of drought stress, both lines responded with increased ABA content; however, the mutant line had a threefold greater level to that of the control.

The difference was still significant at day 6 even if the ABA content decreased in both tomato lines but the lcy-e mutant consistently had higher levels than the Red Setter genotype. At day 15, the “recovery” phase, the ABA level returned to levels similar to that recorded before the start of stress application (day 0) for both genotypes.

### 3.3. Second Drought Stress Experiment

#### 3.3.1. Digital Imaging Results

The second drought stress experiment took place in autumn, as described in Materials and Methods. The response of mutant and wild type plants to the drought stress was monitored through digital RGB images captured at 3, 6 and 12 days after the stress imposition.

**Figure 5:**
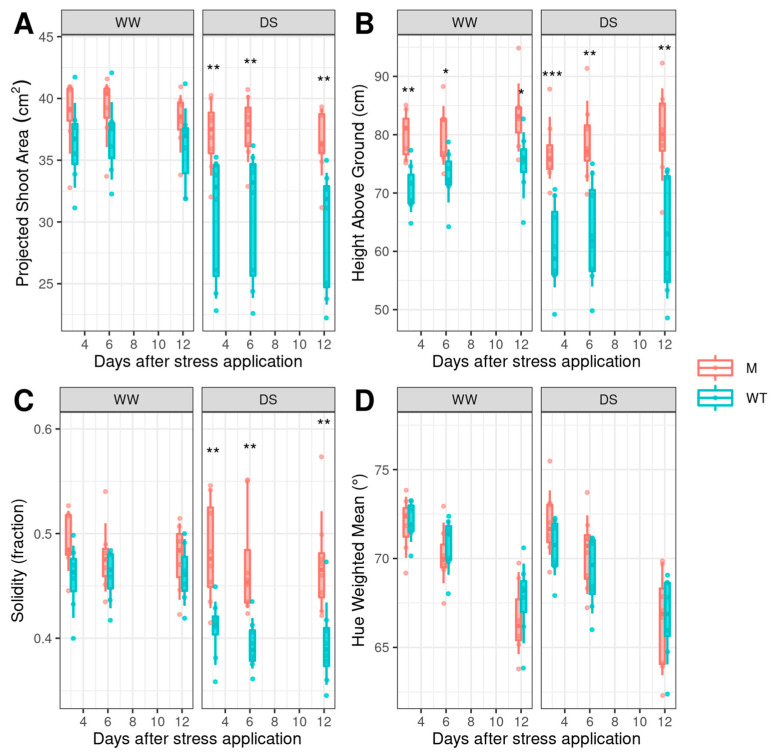
Indices derived from the digital image-based analysis on well-watered (WW) and drought stressed (DS) lcy-e (M) and Red Setter (WT) plants. (**A**) projected shoot area (digital biovolume), (**B**) plant height, (**C**) solidity, (**D**) green index. Values are the mean of six biological replicates ±SD. A one-way ANOVA was used to examine the differences between genotypes at each timing point; the differences among means were identified by Tukey Post-Hoc Test; asterisks indicate significance (* *p* < 0.05, ** *p* < 0.01 *** *p* < 0.001).

As shown in Figure 5, the biomass (projected shoot area) of mutant plants was greater than that of the Red Setter wild type plants; this was the case in both well-watered and drought stress conditions, with the differences being more evident under drought stress.

A similar trend was observed in the plant height trait. In fact, the WW and DS mutant plants were higher than WW and DS wild type Red Setter. The difference between the two tomato lines increased under drought stress.

**Figure 6:**
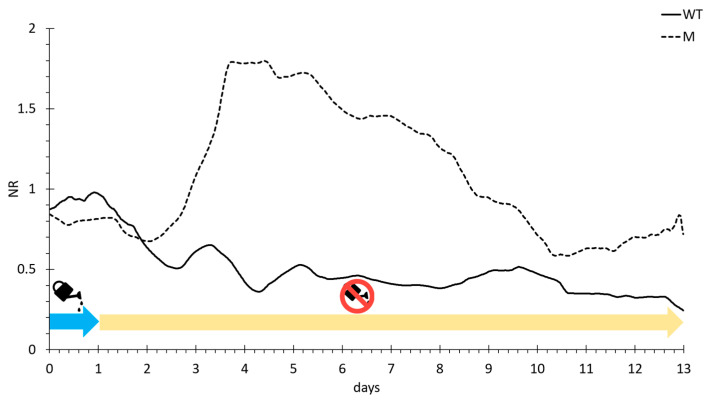
Plot of the daily smooth average of the Normalized sensor Response (NR) measured for 14 days on lcy-e mutant plants (M, dashed line) and Red Setter wild type plants (WT, solid line). Blu arrow indicates full irrigation, yellow arrow indicates drought stress imposition.

The different response of mutant and wild type plants to drought was also shown by the solidity index. Solidity indicates how much of the plant hull area is covered by leaves and is the ratio of plant pixel area to the pixel area of the convex hull shape containing all of the plant pixels. A diminished solidity could therefore be caused by increasing the convex hull area while keeping the plant area pixels constant, or decreasing the plant area pixels while keeping the convex hull area constant. As shown in Figure 6, solidity of WW wild type Red Setter and lcy-e mutant plants was not significantly different, but under drought stress the wild type plants displayed a lower solidity with respect to the mutant. The lower solidity can be explained as a consequence of the reduced plant pixel area and suggests greater wilting of the wild type leaves with respect to the lcy-e mutant.

#### 3.3.2. Bioristor Results

Six drought stressed and six well-watered tomato plants of lcy-e and Red Setter genotypes were each monitored in vivo with a bioristor for 14 days. The trend of the Normalized Sensor Response (NR) was monitored continuously and recorded for the entire length of the experiment (Figure 6).

A variation in the NR of the wild type occurred at day 1 and a rapid and continuous decrease of NR was registered with two typical drought avoidance peaks at day 3 and 4. At day 4, the sensor response reached a steady level that continued until the end of the drought stress imposition (Figure 6).

An opposite trend was observed for the NR of the mutant plants, which showed a slight decrease after 12 h from the beginning of the stress and then the slope showed a rapid and continuous increase up to day 4 reaching maximum values, leading to the hypothesis that the accumulation of osmolytes and compounds was triggered by a defense response. From day 4 to day 10, the NR trend rapidly decreased indicating a stress condition, reaching at day 10 a NR value comparable with pre-stress. From day 10 to the end of the experiment, there was a slow steady increase in NR for the mutant line.

The Analysis of Variance (ANOVA), performed on R values of WW and DS plants for both treatments to consider all possible variables, showed a high significance difference between WW and DS plants (*p* ≤ 0.001) throughout the stress period of the experiment.

**Figure 7:**
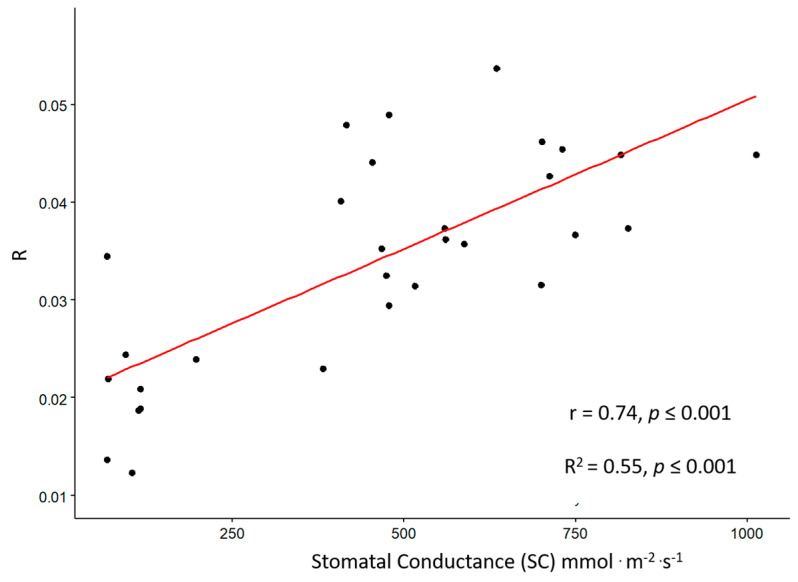
Scatter plots of the sensor response (R) and stomatal conductance (SC) measured on well-watered and drought stressed plants of lcy-e and Red Setter genotypes. The scatter plot and the displayed linear regression indicate a strong correlation between the two variables, with a correlation coefficient of r = 0.74. *p* ≤ 0.001 indicates the statistical significance level of the observed correlation.

Physiological and digital-image base phenotypic traits were compared with those acquired using bioristor. It should be noted that data physiological and phenological traits are measured at time points, while bioristor measurements continuously measure physiological changes in the plant sap and are potentially more sensitive to dynamic variations. The stomatal conductance (SC), investigated as physiological trait strongly affected by drought stress, was recorded and a correlation analysis was carried out with the data collected by bioristor. A strong, highly significant correlation was observed between sensor response (R) and stomatal conductance (SC) (r = 0.74, *p* ≤ 0.001; Figure 7).

The bioristor measurements were correlated with RGB indices acquired through the Scanalyzer 3D platform: (i) plant height, (ii) plant solidity, (iii) digital biovolume and (iv) hue circular (Figure S2). Notwithstanding the applied drought stress, the plants’ growth as indicated by their digital biovolume (projected shoot area), height and solidity, was not strongly affected by the water scarcity.

**Figure 8:**
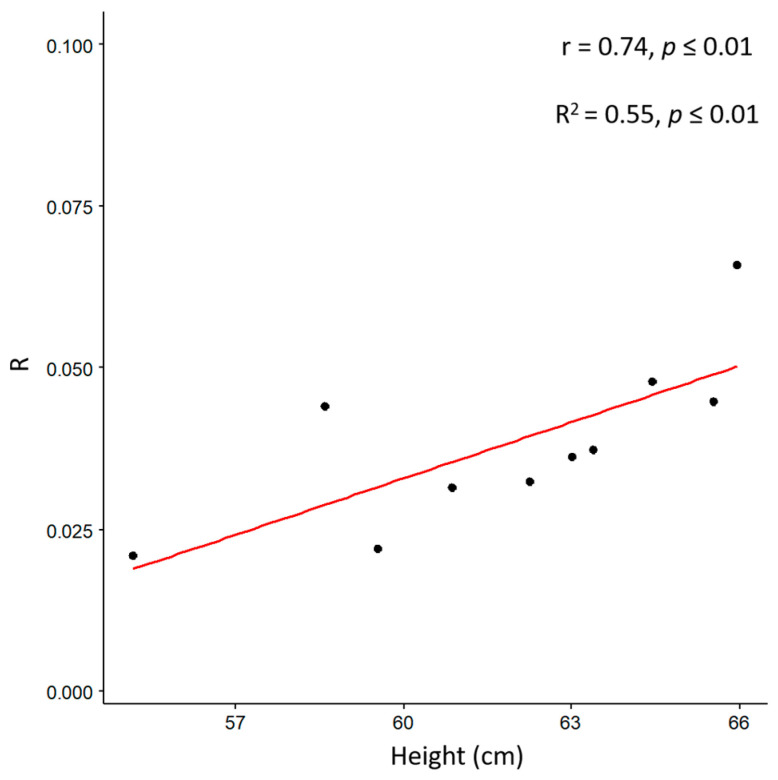
Scatter plots of the sensor response (R) and height (cm) measured on well-watered and drought stressed plants of mutant and wild type genotypes. The scatter plot and linear regression displayed indicate a strong correlation between the two variables, with a correlation coefficient of r = 0.74; *p* ≤ 0.01 indicates the statistical significance level of the observed correlation.

R was correlated with the plant height (r = 0.74, *p* ≤ 0.01, Figure 8), and there was a good correlation with digital biovolume as previously observed. The ability of a bioristor to monitor the in vivo changes occurring in the plant sap, correlated with changes in morphophysiological traits, was supported here.

**Figure 9:**
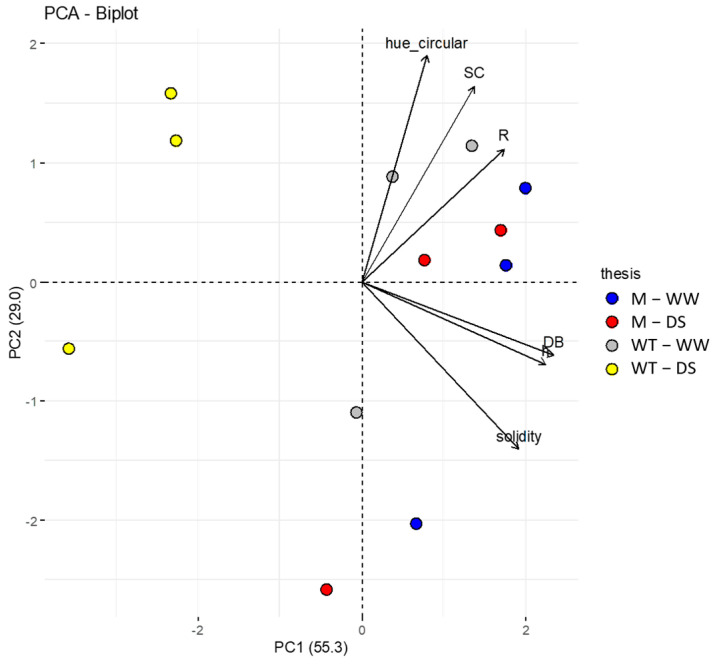
Biplot showing the PCA results. The first two PCs display 84.3% of the total phenotypic variation observed. The component scores (shown in points) are colored according to the combination of genotype and grown conditions. Blue, well-watered (WW) mutant plants (M); red, drought stressed (DS) mutant plants (M); grey, well-watered (WW) wild type plants (WT); yellow, drought stressed (DS) wild type plants (WT). The component loading vectors (represented in lines) were superimposed proportionally to their contribution. SC: stomatal conductance; R: sensor response; DB: digital biovolume; h: plant height; hue_circular; solidity.

As shown in Figure 9, a PCA analysis was performed considering physiological, phenotypic traits and the sensor response (R). The first two components (PC1 and PC2) explain 84.3% of the variability of the described phenomenon. The first PC (PC1) explains 55.3% of the phenotypic variation, and perfectly separates the genotypes and the different treatment. The wild type DS plants are separated in the biplot from all other genotypes, indicating that drought stress strongly impacted on all traits analyzed.

The presence of the lcy-e mutant genotype within the Red Setter well-watered group support the role of the novel G/3378/T LCY-E allelic variant in conferring drought tolerance. R and stomatal conductance are strongly and positively correlated (r = 0.74, *p* ≤ 0.001 as previously demonstrated by Janni et al. [22] and Kim et al. [34,35].

## 4. Discussion

Plants commonly experience periods of drought during their life cycle [36]. Drought stress therefore represents a critical constraint for crop productivity [37,38]. The availability of genetic resources more adaptable to the ongoing climate change and more tolerant to water deficiency with no reduction in their nutritional properties is highly desirable and demanded. Within this context, the molecular screenings of our Red Setter cultivar-based TILLING platform allowed the identification of a novel lycopene ε-cyclase gene (*SlLCY-E*) variant that has the ability to increase the lycopene and total carotenoid content in ripe fruits and to improve abiotic stress tolerance in tomato plants.

The novel *SlLCY-E* allelic variant consists of a missense point mutation that was predicted tolerated by the LCY-E protein activity, according to the SIFT computational analysis [39]; however, the amino acid substitution at position 356 of the lycopene ε-cyclase protein (W356L) was unusual in the set of 12 protein sequences used by SIFT for its prediction. Furthermore the search for any predicted mutations of the *SlLCY-E* gene among the sequenced 360 tomato accessions [40] in the Tomato 360 variants SL2.50 genome browser at SGN (https://solgenomics.net/jbrowse_solgenomics/, accessed on 1 February 2023), accessed for position SL2.50ch12:2288749, revealed that there are no predicted natural mutations at the searched position. This suggests that the TILLING G/3378/T point mutation in the *SlLCY-E* gene is a new and unique mutation. The discovered TILLING missense mutation appears not to be present in the natural variability, therefore we decided to study its influence on the tomato plant phenotype.

As the *SlLCY-E* gene encodes a key enzyme in the carotenoid pathway, the lycopene ε-cyclase, the effects of the missense point mutation on plant phenotype was assessed by analyzing the carotenoid profile and content of leaves as well as ripe fruits of lcy-e mutant plants by comparison to the Red Setter control plants (Table 2).

The observed leaf and fruit lcy-e carotenoid profile is in agreement with the impaired activity of the lycopene ε-cyclase enzyme. It is postulated that the G/3378/T missense mutation affects lycopene ε-cyclase activity with respect to the wild type LCY-E protein and, as a consequence, a decrease in lutein content in leaf and ripe fruit is observed. The deleterious effect of the TILLING missense mutation on lycopene ε-cyclase activity shifts the synthesis of carotenoids towards the β-branch of the pathway, thus increasing the β,β-xanthophyll content in leaf tissue. The impaired activity of the lycopene ε-cyclase also explains the increment of lycopene and consequently of total carotenoids in ripe fruits.

The increase in total carotenoid content observed in lcy-e ripe fruits was not seen in lcy-e leaves of plants grown in greenhouse controlled conditions. A similar result is reported in *Brassica napus* by Bianyun Y. et al. [17] that, by reducing the expression of lycopene ε-cyclase using RNAi, an increase in total carotenoid in seeds of transgenic plants was obtained, but not in the leaves. Differences in capacity to make and store excess carotenoids were proposed to explain the differences between the tissues.

The leaf carotenoid profile of lcy-e i.e., reduction in lutein and increase in β,β-xanthophyll content was still conserved in the mutant line when subjected to drought stress by withholding watering for 15 days. In particular, the violaxanthin content, even if slightly decreased from the sixth day of the stress imposition, continued to be roughly two times higher in lcy-e leaves than in control genotype.

The elevated content of violaxanthin together with neoxanthin, which are proposed to be the abscisic acid precursors, could explain the higher level of ABA measured in the DS mutant line with respect to the DS control line Red Setter.

Surprisingly, no significant difference in ABA content was observed between WW lcy-e and Red Setter plants despite that violaxanthin content in the mutant line was double that in the wild type line. The amount of measured ABA was, in fact, roughly 5 µg∙g^−1^ DW at all the analyzed timing points (Figure 4). In sweet potato plants where the expression of *IbLCY-E* was down-regulated by RNAi, an increased level of ABA content was instead measured under both normal and stress (drought and salt) treatment conditions, in agreement with the higher violaxanthin content of the transgenic plants [18]. In our lyc-e TILLING mutant, the positive correlation between violaxanthin content and ABA concentration was not found and the expected increase of ABA due to the high leaf β,β-xanthophyll concentration was not observed in WW mutant plants.

Perhaps the different response of tomato lcy-e line and transgenic sweet potato, both “affected” in lycopene ε-cyclase activity by means of two different approaches (TILLING missense mutation and down-regulation by RNAi respectively), could be attributed to a diverse mechanism regulating the ABA level and biosynthesis in the two plant species.

The lack of a strict correlation between violaxanthin content and ABA concentration was also reported for the tomato mutant line hp3 (high pigment 3) by Galpaz et al. [41]. Due to the deleterious effect of a missense mutation occurring in the gene for zeaxanthin epoxidase (Zep), which converts zeaxanthin to violaxanthin, hp3 leaves have low traces of violaxanthin and neoxanthin, the two β,β-xanthophyll precursors of ABA. Concerning this mutant line, the authors report that hp3 plants grown in the greenhouse under well-watered conditions showed a low level of ABA but similar to that of wild type plants grown under the same condition, despite their small leaf β,β-xanthophyll content. A significant decrease in ABA levels was instead detected in field-grown hp3 plants compared to the wild-type. These results, i.e., absence and presence of differences in ABA content between hp3 mutant and wild type genotype, are identical to our findings with lcy-e and Red Setter plants grown in well-watered and drought stress conditions respectively.

Several studies have described the damaging influences of drought stress on the vegetative growth of plants [42]. Investigations performed in greenhouse [43] and field [44] conditions demonstrated that DS affects tomato plant growth by reducing plant height, shoot length and number of leaves per plant. Our studies showed that drought stressed lcy-e plants grow much better than the Red Setter control plants, as revealed by their high values of biovolume and height, measured with the digital image-based analysis. The lcy-e tolerance to drought stress is also shown by the lower wilting of the mutant leaves with respect to the wild type genotype, as indicated by the solidity index.

The integration of bioristor data with image-based phenotyping has provided insights into the dynamics and timing of the tomato lcy-e line phenotypic and physiological response. The analysis of the bioristor data suggests that, as an initial response to the drought stress, a change in the transport, allocation, and production of metabolites and ions occurs within the plant, which acts as a signal for stomatal closure and the subsequent decrease in transpiration [22,45].

The high correlation coefficient between the sensor response (R), stomatal conductance (SC), and height trait confirms the hypothesis that the bioristor can detect ions and molecules related to the drought stress and those dissolved and transported through the transpiration stream, thus efficiently detecting the occurrence of drought stress immediately after the priming of the defense responses. A difference in the extent and timing of a possible drought avoidance was observed between the two tested tomato genotypes, together with the different responses to water stress monitored between lcy-e mutant and Red Setter wild type plants.

The combination of the digital image-based data and the bioristor values in a PCA, allowed the clear separation of stressed and unstressed plants. The stressed lcy-e plants clustered with the Red Setter well-watered group, supporting the role of the G/3378/T *LCY-E* allelic variant in conferring drought tolerance. These findings open new perspectives for the use of the bioristor as a tool to study and select drought tolerant genotypes and varieties.

The overall results of the present study demonstrate that the TILLING G/3378/T *LCY-E* allele is a valuable genetic resource that can be used for developing new tomato lines improved in drought stress tolerance and in fruit lycopene and carotenoid content.

Since it has been identified in a processing tomato cultivar (Red Setter), the G/3378/T *LCY-E* allele can be directly used in breeding programs or incorporated in the genetic backgrounds of interest. Open field evaluation conducted on the lcy-e and Red Setter lines under regular irrigation confirmed that the G/3378/T missense mutation favors the accumulation of lycopene and total carotenoids in the fruit. In addition the results of the field trial showed that the new allelic variant of the *SlLCY-E* gene does not affect the most important technological parameters of fruit, such as fruit dimension, fruit weight, firmness and soluble solid content.

Altogether, our phenotypic data prove the usefulness of G/3378/T LCY-E allele in creating new tomato lines addressed to industry or fresh markets.

## 5. Conclusions

Through TILLING screenings of our Red Setter tomato mutant population we identified a novel lycopene ε-cyclase (LCY-E) allele (G/3378/T), producing an increment of lycopene and total carotenoid content in ripe tomato fruit and an increase of β,β-xanthophylls in leaf tissue.

Under water deficiency conditions, the new G/3378/T LCY-E allele produces more ABA than the Red Setter control genotype and it is more tolerant to drought stress, as revealed by the in vivo phenotyping via OECT sensor, which detected a higher changes of ion concentration in the sap of the mutant than in wild type and digital imaging analysis.

Our phenotypic data demonstrate that the G/3378/T LCY-E allele is a valuable genetic resource, useful for developing new tomato varieties with improved drought stress tolerance features and enriched in fruit lycopene and carotenoid content.

## Figures and Tables

**Table 1:** List and sequences of the primers employed in the TILLING molecular screening.

Primer	Sequence 5′-3′
Fw-ext	TCAGACACGACGCTCAATCT
Rev-ext	TGTCGTTTTCGTTCTTGTGG
Fw-int	CCAACACGAGTCTTTTTCGAG
Rev-int	AGTACAGAGGCGCATTTTGG

**Table 2:** Leaf and Fruit carotenoid content (µg·g^−1^ DW) of lcy-e (M) and Red Setter (WT) plants.

	Violaxanthin	Zeaxanthin	Neoxanthin	Lutein	β-Carotene	Lycopene	Total Carotenoids
Leaf							
M	288.51 ± 53.74 ^a^	31.95 ± 3.86 ^a^	101.25 ± 22.37 ^a^	396.55 ± 56.82 ^a^	444.33 ± 31.96 ^a^		1262.6 ± 98.16 ^a^
WT	144.18 ± 18.35 ^b^	22.05 ± 2.79 ^b^	101.31 ± 15.66 ^a^	615.02 ± 14.08 ^b^	395.19 ± 10.49 ^a^		1277.74 ± 36.4 ^a^
Fruit							
M				7.2 ± 1.20 ^a^	37.27 ± 6.26 ^a^	1819.28 ± 104.53 ^a^	1874.5 ± 110.33 ^a^
WT				20.59 ± 2.04 ^b^	54.62 ± 7.86 ^b^	1474.40 ± 51.08 ^b^	1571.18 ± 49.08 ^b^

Data are presented as mean ± SD. Each mean was derived from determinations carried out on three biological replicates. Means with the same letter are not statistically different (Tukey test, *p* < 0.05). M indicates the mutant line lcy-e, WT the control line Red Setter.

## Data Availability

The data presented in this study are available in the article and supplementary material.

## Supplementary Materials

The following supporting information can be downloaded at: https://www.mdpi.com/article/10.3390/genes14061284/s1, Figure S1: Schematic representation of the carotenoid pathway; Figure S2: Pearson’s Correlation (r) of the bioristor response (R) and image-derived data.
