# Supplementary material for: A Lycopene ε-Cyclase TILLING Allele Enhances Lycopene and Carotenoid Content in Fruit and Improves Drought Stress Tolerance in Tomato Plants
Source: Genes (Basel). 2023 Jun 17;14(6):1284. doi: 10.3390/genes14061284 (PMC10298496; doi:10.3390/genes14061284)
Supplement: Supplementary file 1 [file genes-14-01284-s001.zip › Figure S1.pdf]

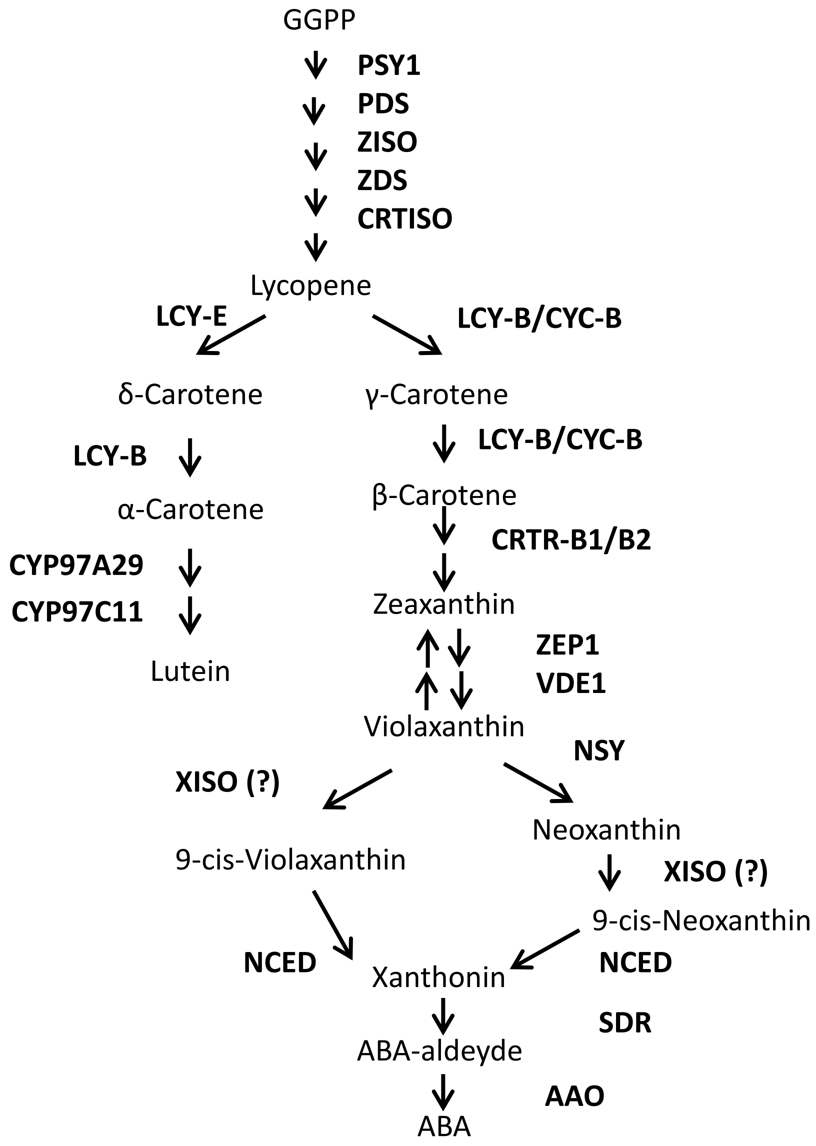

Figure S1. Schematic representation of the carotenoid pathway. Enzymes involved in each step are highlighted in bold. PSY1, phytoene synthase; PDS, phytoene desaturase; ZISO, 15-cis-z-carotene isomerase; ZDS, z-carotene desaturase; CRTISO, carotenoid cis-trans-isomerase; LCY-B, lycopene β-cyclase; CYC-B, lycopene β-cyclase; LCY-E, lycopene ε-cyclase; CRTR-B1/B2, β-carotene hydroxylase; CYP97A29, Cyt P450-type monooxygenase 97A29; CYP97C11, Cyt P450-type monooxygenase 97C11; ZEP1, zeaxanthin de-epoxidase; VDE1, violaxanthin desaturase; NSY, neoxanthin synthase; XISO, xanthophyll 9-cis isomerase; NCED, 9-cis-epoxycarotenoid dioxygenase; SDR, short-chain dehydrogenase/reductase; AAO, abscisic aldehyde oxygenase.
