# Supplementary material for: A Lycopene ε-Cyclase TILLING Allele Enhances Lycopene and Carotenoid Content in Fruit and Improves Drought Stress Tolerance in Tomato Plants
Source: Genes (Basel). 2023 Jun 17;14(6):1284. doi: 10.3390/genes14061284 (PMC10298496; doi:10.3390/genes14061284)
Supplement: Supplementary file 1 [file genes-14-01284-s001.zip › Figure S2.pdf]

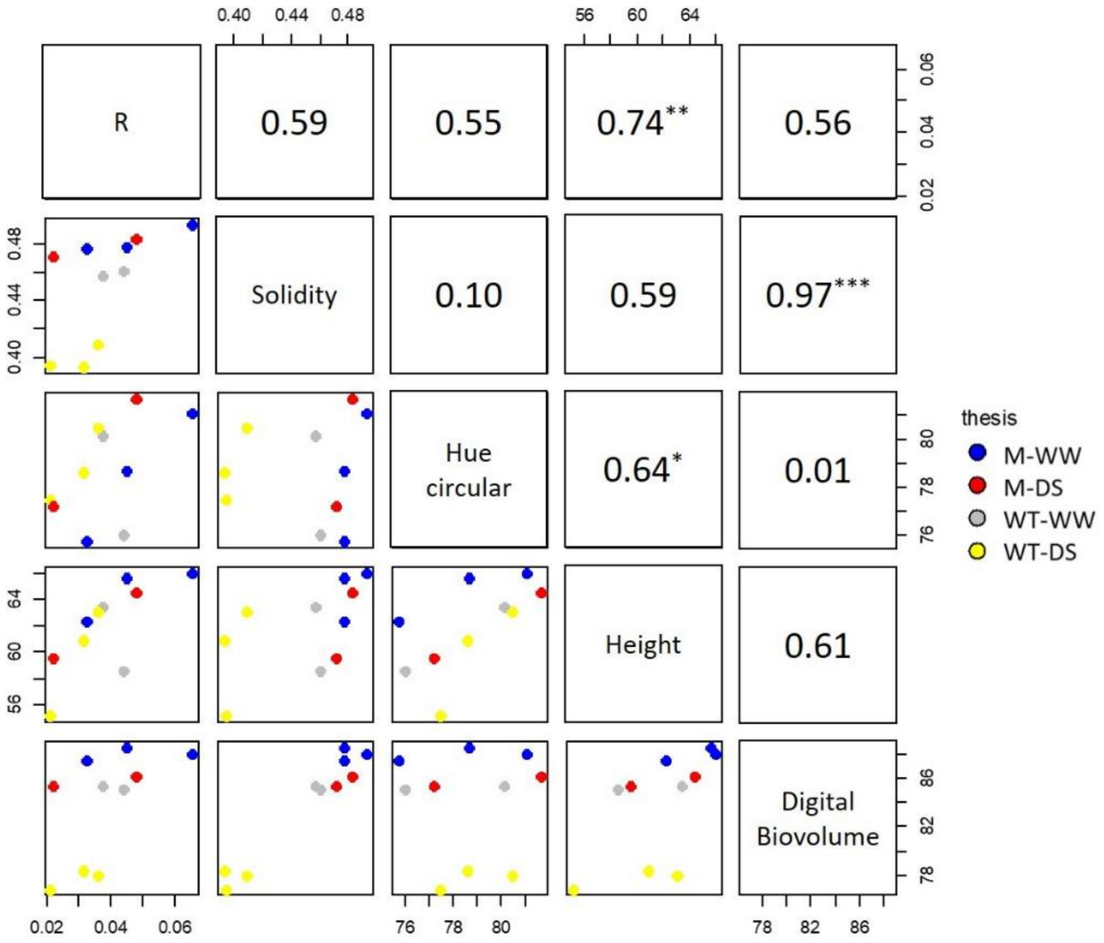

Figure S2. Pearson's Correlation (r) of the the bioristor response (R), and image-derived data (Solidity, Hue circular, Plant height and Digital Biovolume). Blue dots, well-watered (WW) mutant plants (M); red dots, drought stressed (DS) mutant plants (M); grey dots, well-watered (WW) wild type plants (WT); yellow dots, drought stressed (DS) wild type plants (WT). Asterisks indicate the significance levels of the correlation coefficients ($p \leq 0.01$, $**$; $p \leq 0.05$, $*$).
